# Ozonated Oils as Antimicrobial Systems in Topical Applications. Their Characterization, Current Applications, and Advances in Improved Delivery Techniques

**DOI:** 10.3390/molecules25020334

**Published:** 2020-01-14

**Authors:** Elena Ugazio, Vivian Tullio, Arianna Binello, Silvia Tagliapietra, Franco Dosio

**Affiliations:** 1 Department of Drug Science and Technology, University of Torino, 10125 Torino, Italy; elena.ugazio@unito.it (E.U.); arianna.binello@unito.it (A.B.); silvia.tagliapietra@unito.it (S.T.); 2 Department of Public Health and Pediatrics, Microbiology Division, University of Torino, 10126 Torino, Italy; vivian.tullio@unito.it

**Keywords:** ozonated oils, topical anti-infectives, vegetable oils, delivery systems

## Abstract

The search for a wide spectrum of antimicrobial agents that can avoid resistance while maintaining reasonable side effects has led to ozonated oils experiencing an increase in scientific interest and clinical applications. The treatment of vegetable oils with ozone leads to the creation of a reservoir of ozone that slowly releases into the skin thanks to the fact that ozone can be held as ozonides of unsaturated fatty acids. Interest in the use of ozonated oils has meant that several ozonated-vegetable-oil-containing products have been commercialized as cosmetic and pharmaceutical agents, and in innovative textile products with antibacterial activity. New approaches to the delivery of ozonated oils have very recently appeared in an attempt to improve their characteristics and reduce drawbacks, such as an unpleasant odor, high viscosity and undesired effects on skin, including irritation and rashes. The present review focuses on the current status of delivery agents that use ozonated oils as antimicrobial agents in topical (dermal, skin, and soft tissues) treatments. Challenges and future opportunities for these delivery systems will also be discussed.

## 1. Introduction

Research interest in “natural” medicine has greatly increased over the last fifty years and, above all, plant products have seen widespread use in the microbiological field. While there are a number of new therapeutic approaches that are based on medicinal plants and their extracts, the use of oils is attractive and increasingly under investigation. Interest in oils has recently revived and they have started to be recognized for their potential antimicrobial role. This new trend is due both to the increasing spread of microorganisms that are resistant to conventional antimicrobial agents, and to the increase in studies on the antimicrobial activity of natural products. Drug resistance can arise when bacteria and yeasts “mutate” rapidly, changing their cellular membrane proteins so they are no longer recognizable by drugs. In fact, most bacteria are able to modify their PBPs (a group of bacterial cellular membrane proteins that are characterized by their affinity for and binding of β-lactam drugs) or produce, for defensive purposes, several enzymes, such as endo and eso-β-lactamases or transferase that are able to inactivate β-lactam and aminoglycosides antibiotics, respectively. In yeasts, resistance to antifungal agents, especially to azoles, can arise because of changes to efflux pumps or a drastic reduction (50%) in ergosterol levels in cellular membranes, making it unavailable for use as a drug target site [1,2,3].

In addition to these problems, antibiotics used against pathogen microorganisms can induce severe side effects, especially in patients that are undergoing prolonged therapeutic treatment, and may also alter the microbiome, which is important for the eubiosis of the intestines and body as a whole. Antimicrobial agents that increase dysbiosis create an ideal environment for pathogen microorganism colonization and further infections with possible recurrent episodes [4,5]. Conversely, most natural products, which contain hundreds of naturally active ingredients in variable proportions, eliminate the risk of antibiotic resistance as microbes are not able to adapt to their heterogeneous structures. For this reason, many natural compounds, in particular some vegetable oils such as olive oil (considered to be one of the most excellent foods), are also currently being tested with a view to microbiological action to assess their possible use in the anti-infective field.

The therapeutic and health properties of olive oil have been known for millennia, so much so that Hippocrates advised the use of fresh olive juice to cure mental illness and in wraps to heal ulcers. During the Middle Ages and the Renaissance, olive oil was used to cure gynecological infections and was considered useful in the treatment of heart disease, fever, and hypertension. From a dermatological point of view, olive oil has also proven to have antimicrobial activity in infections of burnt skin, against Gram-positive bacteria, such as *Staphylococcus aureus*, Gram-negative bacteria, and various species of fungi, including *Candida* spp. [6,7]. In fact, these properties make olive oil an important component of some topical formulations that are used in the treatment of inflammatory and mycotic skin diseases [6]. However, it has been demonstrated that it is possible to further enhance the properties of oils by adding ozone. Indeed, ozone (O_3_) has been recognized as one of the best bactericidal, antiviral, and antifungal agents, and has been used empirically as a clinical therapeutic agent [8,9]. The mechanisms behind this activity are based on the greater oxidizing properties of ozone, which induce the destruction of bacterial cell walls and the cytoplasmic membrane. The result is an increase in permeability and the entry of ozone into bacterial cells [10]. Moreover, ozone is also able to stimulate the innate immune system to contrast microorganisms. In fact, in the presence of ozone, microorganism lipoproteins produce LOP (products of lipid oxidation) that are able to induce higher amounts of H_2_O_2_, by phagocytes, resulting in better bacteriostatic and bactericidal activity [11,12]. In order to improve the short half-life of oxygenated agents (ozone water, ozone dimethylsulphoxide, etc.), gaseous ozone has been used in chemical reactions with unsaturated substrates, producing ozonated derivatives, particularly oils and butter of vegetable or animal origin. While the first description of the chemical reaction was reported by Fenaroli in 1906 [13], the antifungal properties and properties as healing and soothing remedies for skin with olive-oil derived ozonides was described in the 1930s [14]. Ozonated vegetable oils have since been evaluated for their antibacterial and antifungal activity [15,16], their use in topical skin treatments (dermatology) and particular efficacy in the healing of cutaneous wounds, and these studies have increased in number in recent years [12,13,14,15,16,17]. Consequently, the number of articles and patents on the improved behavior of ozonated oils using delivery approaches mean that they are more frequently being suggested as promising materials for cosmetics and pharmaceutical therapies. The present review describes, in detail, the chemical aspects of ozonated oil, such as the ozonation process, in order to evaluate the most relevant aspects that have an impact on final product quality and to provide complete information about ozonated oils and their potential applications. Moreover, the analytical characterization and stability of the products are discussed. Furthermore, updated evaluations on the major topical applications of ozonated oils as antimicrobial agents are presented. The last section focuses on data on the current rationale of ozonated oil delivery systems. In conclusion, a comparison with other micro/nano delivery approaches and some considerations and suggestions for future development are presented.

## 2. Ozonation Process

Ozonated oils are obtained using ozone generators, and several parameters can affect the quality of ozonated derivatives. When approaching ozonation procedures, one must consider several parameters: The quality and efficacy of ozone generators; ozonation conditions, such as time, ozone concentration and flow, temperature and the agitation of the reaction mixture; vegetal oil type and quantity, and the presence of water or other catalyzer. Moreover, the use of medical grade oxygen may be a possible means to avoid the production of potentially toxic nitrated by-products, formed via the nitrogen content in air, with a collateral decrease in ozonation efficiency [18]. Medical oxygen has been reported to be highly efficient, by Díaz et al. in sunflower oil ozonation [19]. It was reported that air and medicinal oxygen, for 8 and 5 h respectively, gave very similar peroxide index values (about 660 mmol/kg) and no decreases in the antimicrobial activity of the ozonated oils was observed.

As ozone reacts with carbon–carbon double bonds, a higher degree of unsaturation leads to longer ozonation reaction times [20]. This factor clearly influences ozonation kinetics, as a function of vegetal oil origin and their fatty acid composition, especially when sunflower oils, olive oils, and, even more so, “classical” or “high oleic” sunflower oils are subjected to this oxidation procedure. In fact, classic sunflower oil is mainly composed of linoleic acid (18:2) and requires less ozonation time than the high oleic analogue, which contains about 90% oleic acid (18:1) and is therefore more similar to olive oil.

### 2.1. Ozonation Mechanism

Ozone is a powerful oxidizing agent that can react with the unsaturated triacylglycerides present in vegetal oils. The mechanism proposed by Criegee in 1975, involving the oxidative cleavage of double bonds, has been widely accepted [21]. The products were indicated as aldehydes and ketones, their peroxidic derivatives and the carboxylic acids deriving from aldehyde-compound oxidation. 

**Scheme 1:**
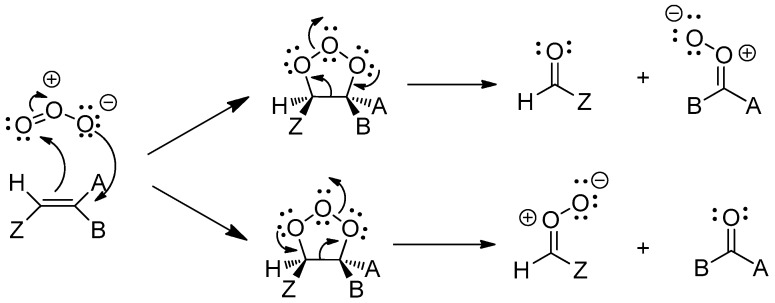
Primary ozonide generation and decomposition to carbonyl oxide and carbonyl compounds.

Briefly, the ozonation mechanism proceeds via discrete steps, including 1,3-dipolar cycloadditions and their reversion. The process starts with the generation of the primary ozonide (also called molozonide, 1,2,3-trioxolane, or Criegee intermediate), which decomposes to carbonyl oxide and a carbonyl compound, as described in Scheme 1.

**Scheme 2:**
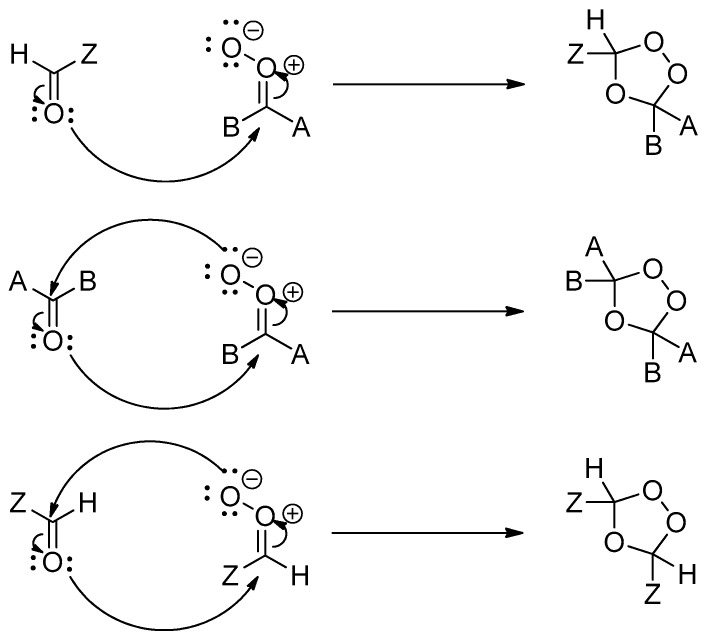
Secondary ozonide generation.

The carbonyl oxides lead to a mixture of more stable 1,2,4-trioxolanes (secondary ozonides), via a 1,3-dipolar cycloaddition to the carbonyl compounds, involving reverse regiochemistry (Scheme 2).

**Scheme 3:**
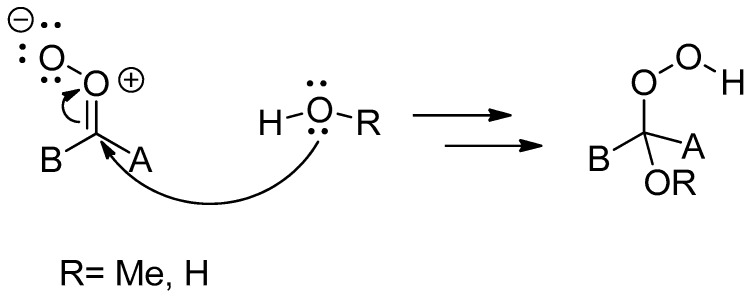
Reaction of carbonyl oxide with protic solvent.

When ozonation is carried out in the presence of protic solvents, such as alcohol or water, the carbonyl oxide can generate hydroperoxy hemiacetals or mono hydroperoxy gem diol (Scheme 3).

**Scheme 4:**
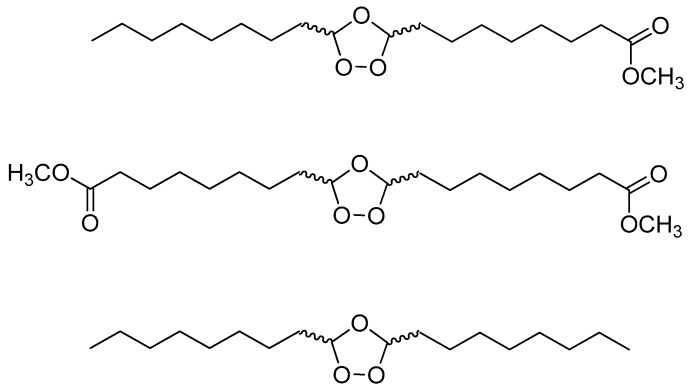
Secondary ozonides generated by ozonolysis of methyl oleate (stereochemistry not assigned).

If we look at the stereochemistry implications of this kind of process, we can see that the first step of ozonolysis, which leads to primary ozonide generation, is stereospecific. The stereoselectivity arises over the subsequent steps. Kuczkowski has summarized this stereoselectivity and observed that the ozonolysis of cis(trans)-1,2-alkenes with bulky substituents leads to a higher yield in cis(trans)-secondary ozonides [22]. The ratio of cis and trans 1,2,4-trioxolanes is therefore not only a function of ozonation conditions, but also related to the double bond stereochemistry and substituent steric effect. The complexity of the lipid mixture in vegetal oils makes it difficult to clarify the structural and isomeric composition of the generated secondary ozonides, also when methyl oleate is chosen as the fatty acid model [23]. A total of six isomeric ozonides (cis and trans normal forms, and two types of cross-ozonides) have been distinguished and identified by Wu et al. [24] (Scheme 4), which should be considered when the possible therapeutic effects of ozonated oils are evaluated.

Thanks to the use of spectroscopic techniques, Georgiev et al. have found that the trans isomer is predominantly formed in olive oil ozonides, with a cis/trans ratio of 46/54 [25].

The percentage efficiency of ozonation processes can be calculated as the ratio between the peroxidation amount, estimated by the peroxide value, and the total amount of applied ozone as indicated in the following equation [26]:

(1) $$\mathrm{Ozonization}\;\mathrm{efficiency}=\frac{\left(\mathrm{PVs}-{\mathrm{PV}}_{0}\right)}{1000}\times \frac{O_{3}\mathrm{EW}}{\mathrm{AOD}}\times 100$$

where PV_S_ is the peroxide value of the ozonated oil, PV_0_ is the peroxide value of the oil before its ozonation, O_3_EW is ozone equivalent weight, and AOD corresponds to the applied ozone dose (mg/g).

Moreover, it is important to evaluate the ozonation degree of the oil, as a higher oxidation level does not always correspond to a greater therapeutic effect. For example, Guerra-Blanco et al. have observed that major anti-inflammatory and wound-healing effects were provided by ozonated grape seed and sunflower oils with a low ozonation degree (22%–24%) [27].

### 2.2. Ozone Absorption

Ozone absorption represents the amount of ozone consumed by oils during ozonation, and can be calculated from the difference between the amount of ozone produced during the procedure and the amount of non-reacted ozone, which is determined by iodometric titration. Briefly, the free iodine formed by the reaction of ozone with iodide is titrated with sodium thiosulfate, and the rate of ozone production is evaluated by making it directly absorb into an acidic potassium iodide solution in a blank experiment [28].

A qualitative and quantitative determination of ozone amount in oil samples (e.g. sunflower, castor, olive, and almond oils) can be carried out by taking advantage of the reaction between ozone and indigo trisulfonic [29]. It is a colorimetric method based on the fact that the reaction when the two reagents are combined leads to the formation of colorless products, meaning that the change in the intensity of the dye color non only indicates the presence of ozone, but is also proportional to the ozone amount in the oil sample.

## 3. Characterization of Ozonated Oils

The above-mentioned oxygenated compounds that are produced through ozonation may be responsible for the wide biological activity of ozonated vegetable oils. In order to give an objective meaning to the term “ozonated” when related to a vegetal oil, it is important to quantify the amount of peroxides in the considered sample. In a pharmacological context, ozonide amount is an important measure of the capacity of the oil to deliver active O_2_ and other active species that can be exploited in the treatment of skin diseases [18]. It follows that knowledge of by-product formation is helpful in evaluating the ozonation degree required to achieve the desired therapeutic effects.

The quality of the obtained ozonated oils can be assessed by measuring a number of physicochemical parameters [30], that are important for characterization and identification.

### 3.1. Total Unsaturation

A measurement of total unsaturation is a useful control method for monitoring the reaction of ozone with vegetal oils and to evaluate their clinical effectiveness, which is related to the oil’s properties and ozonation degree.

The amount of substrate that can be oxidized with ozone can be quickly (1–3 min) evaluated using this quantitative technique. In particular, the ozone required for the reaction with the oil sample is measured, and then compared with the amount that is consumed during an oxidation reaction carried out with a standard compound of known chemical structure and concentration. In this way, it is possible to have a measure of the oxidizable substrate as a function of the amount of double bonds that are present in the considered vegetal oil. It takes into account not only the fatty acids, but also other minor compounds, such as phenols, vitamins, and pigments, that are in the sample. The total unsaturation value is determined as the mol of double bonds per gram of sample. It was found that more ozonable structures were contained in grape seed oil than in sunflower oil in a comparison of total unsaturation value [31].

### 3.2. Iodine Value

Oil unsaturation rate can be determined using iodine values, which are defined as the amount (grams) of iodine that reacts with the double bonds of oil samples. Measured according to the procedure published by the American Oil Chemists’ Society [18,32] and Pharmacopeia monographs [33], it is a chemical analysis method (a titration with a thiosulphate solution) that allows the double bond decrease and then 1,2,4-trioxolane formation during oil ozonization to be evaluated.

This value is calculated according to the following equation:

(2) $$\mathrm{Iodine}\;\mathrm{value}=1.269\times \frac{\left(n_{1}-n_{2}\right)}{m}$$

where n_1_ represents the mL of thiosulphate solution used in the blank test, n_2_ corresponds to the mL of thiosulphate solution used for the titration, and m is the grams of oil used. While different oil fatty-acid compositions can influence the obtained iodine value, the presence of water during the ozonation processes does not seem to have any impact on the double-bond consumption rate [20]. It is worth noting that high ozonated oil viscosity can obstruct the iodine reagent’s access to double bonds, and can thus cause the iodine value from this assay to be unreliable [34].

### 3.3. Peroxide Value

The peroxide value indicates the amount of peroxide in the ozonated oil and is expressed as the active oxygen amount per kilogram of sample (mmol-meq/kg) [18]. In addition, this simple and rapid method is also used to evaluate ozonated vegetable oil stability, to control its storage conditions and to correlate its potential antimicrobial activities, as a means to monitor the overall ozonization process [35]. The official Pharmacopoeia monograph [36], the American Oil Chemists’ Society procedure [37] and other different methods reported in literature [30,38] can be used to determine the peroxide value. Regardless, the following equation can be used to calculate the parameter:

(3) $$\mathrm{Peroxide}\;\mathrm{value}=1000\times (V_{1}-V_{0})\times \frac{c}{m}$$

where V_1_ represents the mL of thiosulphate solution used for the titration, V_0_ corresponds to the mL of thiosulphate solution used in the blank test, c is the thiosulphate concentration and m represents the grams of used oil.

By comparing the effect of ozonation on olive and soybean oil, Sadowska et al. [28] have observed that the latter displayed a higher increase in peroxide and acid values after a 7 h process. Moreover, the peroxide index values were found to be higher when the ozonation process was carried out on the various sunflower oils in the presence of water [20]: From 560 to 2680 meq of active oxygen/kg after 7 h of oil ozonation with water, and 397 meq of active oxygen/kg of oil ozonate without water for the same process time. The nature of sunflower oil (classic or high oleic versions) seems to lead to different peroxide values when the ozonization is carried out in the presence of water, while the values are much more similar when the process occurs in the absence of this solvent.

### 3.4. Acidity Value

According to American Oil Chemists’ Society [39], the acidity index of ozonated oils is expressed as the quantity of potassium hydroxide necessary to neutralize the free fatty acids in 1 g of product [18]. As an index of acidity level, this parameter allows the formation of the degradation by-products to be monitored during the oil ozonation process. In fact, the ozonides and aldehydes that derive from the hydrolysis reactions of the oxygenated products increase the acidity of the medium [19]. The acid value is calculated using the following equation:

(4) $$\mathrm{Acidity}\;\mathrm{value}=5.610\times \frac{n}{m}$$

where n represents the mL of titrant, and m corresponds to the grams of oil used.

Moureu et al. [20] have observed an increase in this value with ozonation time. An evaluation of the effect due to the addition of water during the ozonation process allowed the authors to report a slower acid value increase for the sunflower oils that were ozonated without this protic solvent, or ozonated in less than 4 h with water. Although the effect of water, which leads to significantly higher acid values, seemed to be less important than the peroxide values, both of these parameters seemed to have an important correlation with the potential antibacterial activity of the ozonated oils. Moreover, a no strong effect on acid value was found when comparing sunflower oils with different origins.

### 3.5. Formaldehyde Determination

Formaldehyde is a by-product of the vegetal-oil ozonation process. Although it is a compound with antibacterial activity, formaldehyde is also a toxic product, and can affect cellular integrity. Its presence can be detected by a heated reaction with chromotropic acid in solution with concentrated sulfuric acid, which generates a violet-colored solution [29].

The amounts of aldehydes in ozonated oil can also be determined using the p-anisidine value analytical method. According to the American Oil Chemists’ Society [40], this procedure involves the addition of free hydroxylamine and the result is expressed as mmol of aldehydes per gram of sample.

### 3.6. Viscosity

The measurement of ozonated-oil viscosity can be carried out using a viscometer device (e.g. torsional oscillation or vibrating viscometer) in a range of temperatures from ambient to 35–40 °C. For example, Zanardi et al. have reported that a range of viscosities, of up to 900 and 400 mPa s, for ozonated sesame oil were observed as a function of the temperature of the determination, 22 and 35 °C, respectively [30].

This parameter estimates the amount of double bonds present in the oil and, generally, a greater ozonation time can be correlated to higher product viscosity and density. In fact, viscosity provides information about the van der Waals interactions, which increase in correlation to the disappearance of the double bonds that have reacted with ozone. Its value is strictly associated to ozonation kinetics, as the modification of unsaturated acyl chains affects the mobility of the species involved. Moreover, the polymerization that can occur during ozonation processes generates polymeric peroxide, resulting in an increase in viscosity [19]. It follows that this method is a useful means of achieving ozonation-process quality control and of optimizing the time of treatment as a function of the desired ozonated vegetal oil.

Guerra-Blanco et al. [31] have found that more polymeric ozonides are formed in grape seed oil than in sunflower oil, and this was demonstrated using the differences in their viscosities. At the end of an ozonation treatment of 5 h, the viscosity measured for grape seed oil was 2.5 times higher than that of the sunflower sample. Nevertheless, a complete rheological behavior could be relevant for a whole characterization of ozonated oils but unfortunately, to the best of our knowledge, there is a complete lack of this kind of evaluation in the literature.

High viscosity and gel-like appearance have been found in the product (oleogel) obtained from the ozonation of sunflower oil in the presence of water, whereas the oleogel that was generated without this polar solvent did not display these features [41]. Some bibliographic references that indicate values obtained from the characterization of several ozonated oils are reported, as examples, in Table 1.

## 4. Analytical Control of Ozonated Oils

Spectroscopic techniques, such as Fourier-transformed infrared (FT-IR) and ^1^H and ^13^C-NMR, can be involved in determining the quality of ozonated products because secondary oxidation products, such as aldehyde compounds, can lead to cytotoxic and genotoxic concerns [30], meaning that precise and reliable analytical controls are indispensable.

### 4.1. FT-IR Spectroscopy

The ozonation process can be evaluated by monitoring some functional groups through FT-IR. In particular, the decrease in the bands that correspond to C=C and =C–H stretching can be followed together with the increase in the band that corresponds to ozonide C-O stretching; values for sesame oil are respectively: 1654, 3009, and 1105 cm^−1^ [30]. A C-O stretching of 1365 cm^−1^ has been reported for the trans isomer of ozonide in ozonated olive oil [25].

FT-IR data in a comparison of ozonation kinetics in grape seed and sunflower oils by Guerra-Blanco et al. has shown that the latter presented faster double-bond decomposition, while the 1H-NMR technique does not allow for significant differentiation between the two oils, although faster ozonide formation was revealed in grape seed oil [31]. The authors ascribed the possible explanation that the amount of unsaturated compounds that were present in the two oils was different.

The existence of ozone within ozonated olive oil nanoemulsion systems, which were prepared to have small droplet diameters (212.7 nm) using the emulsion inversion point low-energy method, has been verified by the use of FT-IR in the range of 400–4000 cm^−1^ [43]. Chemical changes occurred in fatty-acid chains during the ozonolysis procedure, and, according to the Criegee mechanism, were indicated by the presence of the peaks at 3466, 1745, and at 1104 cm^−1^ (ozonide CO stretching). Moreover, nanoemulsion stability after 30 days of storage at room temperature was demonstrated using this spectroscopic technique.

### 4.2. ^1^H and ^13^C NMR

The peroxide value is commonly used to monitor the ozonation process thanks to its easy execution and low cost. However, the necessity to characterize the ozonated oil has recently meant that ^1^H and ^13^C NMR are used to better understand the kinetics of the reactions involved and their ozonation products. This spectroscopic technique can be used to follow the functional group modification that occurs during the treatment of vegetal oil with ozone. On the one hand, the protons and carbons signals related to double bonds are observed to disappear and, on the other, signals related to 1,2,4-trioxolane appear concomitantly. According to Bailey’s observations [44], the ^1^H NMR signals of the cis isomers of the secondary ozonide in ozonated olive oil appear at lower field intensity (5.18 ppm), while the trans isomers are upfield (5.13 ppm) [25]. Moreover, NMR allows quantitative analyses to be carried out using the integral of the glycerol OCH_2_ protons, which do not vary over the ozonation, as a reference. Sega et al. [26] have assessed that the disappearance of unsaturated species and the subsequent appearance of protons related to cyclic ozonated signals in the ^1^H NMR of ozonated sesame oil is linearly correlated to increases in its peroxide and viscosity values, and to a decrease in its iodine number.

The ozonide structure within the ozonated olive oil nanoemulsion system has been confirmed by ^13^C NMR analyses. In particular, the authors referred that the signal at 104 ppm, which indicates the presence of the 1,2,4-trioxolane carbon ring, did not disappear from the nanoemulsion’s structure after 30 days of storage at room temperature [43].

### 4.3. Gas–Liquid Chromatography

The analysis of saturated and unsaturated fatty acids in vegetal oil can be routinely carried out using gas–liquid chromatography (GLC). Rich in oleic acid, olive oil has fewer double bonds available for ozone than sunflower oil, which presents major linoleic-acid content. However, the number of carbon–carbon double bonds can differ from one type of vegetal oil to another, even when different oils of the same typology are compared (e.g. high oleic and classic sunflower oil), and this can influence the antibacterial activity of the ozonated product.

A gradual decrease in unsaturated fatty acids (18:1,18:2), and a corresponding gradual increase in ozone amount, has been found by Diaz et al. [34] using GLC, that was carried out on the fatty acid methyl ester derivatives of ozonated sunflower and olive oil samples. Some of the species that are generated in the ozonation of triglycerides and the unsaturated fatty acids in vegetal oils can be found in the volatile fraction of the product. In particular, the partial decomposition of peroxidic and hydroperoxidic species leads to the generation of aldehydes and carboxylic acids, with different carbon lengths, that can present low boiling points, a characteristic odor and contribute to the composition of the volatile fraction. The characterization of this fraction of the ozonated sunflower oil Oleozon^®^ was performed via gas chromatography and gas chromatography coupled to mass spectrometry [45]. Using purge and trap (cold or Tenax-TA) methods combined with liquid–liquid extraction, the authors identified hexanal, nonanal, 3-nonenal and malonaldehyde as main the components. This was together with smaller amounts of hexanoic acid, nonanoic acid, heptanal, 2-nonenal, and 3-nonenoic acid.

## 5. Ozonated Oil Stability

The ozonides generated during vegetable oil ozonation are all liquid or semisolid at room temperature and their stability has to be assessed to be adequate under usual storage conditions for commercial purposes.

There is a general lack of data in the literature on the stability of ozonated vegetal oils in commercial formulations, such as Oleozon^®^. These kinds of evaluations have often been carried out using the peroxide value as the dedicated method. A stability of at least 6 months has been found for ozonated sunflower oil Neozone 4000 and cosmetic products that contain ozonated sunflower oil after storage in refrigerated conditions or at room temperature, while a significant decrease in ozonide content has been reported for samples stored for more prolonged times and at higher temperatures (40 °C) [46]. The authors analyzed the degradation of fatty acid ozonides using GC/MS analyses of the volatile fraction of ozonated oils; degradation compounds, such as furyl derivatives, saturated and unsaturated aldehydes and carboxylic acids, were found. Nevertheless, it should be remembered that the presence of light and/or moisture may affect glyceryl oil-based ozonides, thus facilitating several decomposition pathways, the principal of which leads to the formation of aldehydes; objectionable odors due to a rancidity can then develop [26].

A high oleic sunflower oil that was ozonated with water for 4 h has had its stability under different storage conditions monitored in terms of time (from 1 to 12 months) and temperature (form −20 to +37 °C) by Moureu et al. [47]. The peroxide index, acidity value, infrared spectra and GLC profile were chosen as the analytical methods and the initial ozonated oil characteristics under storage at room and higher temperatures showed rapid modification, while product stability of over one year was found at fridge or freezer temperatures. However, the activity of the ozonated sunflower oil against *Streptococcus uberis* remained unchanged despite its chemical degradation, and the authors suggested that this is probably correlated to increases in nonanoic and azelaic acid content that can compensate the disappearance of ozonides.

Thanks to its peculiarity, ozonated olive oil (OZO) has been reported to show greater antimicrobial effects and duration, as it can be stored for around a year at room temperature or up to three or four years in a refrigerator [48].

## 6. Topical Applications of Ozonated Oils

Various ozonated oils that are intended for topical applications are currently available on the market. The most frequently used vegetable oils for the production of ozonated derivatives are olive oil and sunflower oil, although the ozonation procedure for these oils requires a long time and a significant standardization in terms of gas-flow, O_3_ concentration, oil volume, and temperature. Although at least twenty different vegetable oils have been patented over the last few years, defining their relative costs/benefits is still an arduous task [49]. Thyme, sesame, soybean, coconut, hemp seed, grape seed, jojoba, sweet almond, peanut, macadamia, rice bran, avocado, flax seed, pumpkin seed, and safflower seed are some examples of such oils.

An evaluation of the main physicochemical parameters, stability, effectiveness and costs has revealed that sesame oil appears to be the most advantageous for ozonization. Essential oils are also sometimes suitable for this use. Ozonia3000^®^ and Oleozon^®^, ozonated sunflower oils manufactured in Italy and Cuba, respectively, Cocozone oil from coconut oil ozonolysis from Britain, and OOO and O2-ZAP^®^ produced in Canada and the USA, respectively, from olive oil are some examples of commonly available products. At the moment, ozonated vegetable oils are used externally for the management and prevention of various skin diseases [12]. The pathological conditions that benefit from this type of treatment are infectious skin diseases, abscesses and athlete’s foot [50,51,52,53], allergic diseases, such as atopic dermatitis, eczema, urticarial and prurigo [54,55], erythema scaly diseases, such as psoriasis and palmoplantar pustulosis [56,57], wound healing, and ulcer recovery [58].

Furthermore, oils themselves act as moisturizers and protectants, particularly for subjects with impaired skin barrier function. For these purposes, medicinal and cosmetic products that are based on ozonated vegetable oils are commercially available. The therapeutic category includes derivatives (ointment, gel, spray) that can be applied to the skin and to some mucous membranes for dermatological treatment, such as antimicrobials, exudate reducers and wound healing stimulants [15], or for the treatment of acne, herpes, psoriasis, fungal infections, bed sores, and wounds in general [11]. Some in vivo studies have shown that the severe skin lesions that are caused by *S. aureus* and methicillin-resistant *S. aureus* (MRSA) have been healed by therapy with ozonated oil in 1–2 months, which prove its great efficacy, limited side effects and low-cost [59]. Indeed, Sechi et al. [60] have investigated the antimicrobial activity of ozonated sunflower oil (Oleozon^®^) against pathogens, such as *S. aureus*, *Enterococcus faecalis*, *E. faecium*, *S. pyogenes*, *Escherichia coli*, *Pseudomonas aeruginosa*, and various species of *Mycobacterium*. The results were very satisfactory, with MICs of 2.37 to 9.95 mg/mL for mycobacteria, and 1.18 to 9.5 mg/ml for all other bacteria being observed. At first, these MIC values may appear to be too high, but we must consider that vegetable oils are very complex. This good antibacterial activity has recently been confirmed by Serio et al. [61] both in Gram-negative bacterial strains (*E. coli* and *P. aeruginosa)*, and Gram-positive strains (*S. aureus* and *Micrococcus luteus*). It has also been observed that Oleozon^®^ displays good antimicrobial activity towards protozoan parasites, such as *Giardia duodenalis* and *Leishmania major* [62]. An anti-leishmania activity obtained by co-administration of ozonated oil and glucantime was recently reported for cutaneous leishmaniasis treatment in humans [63]. Good effects in repairing lesions of leishmaniasis and even other ulcers have been described, while the only transient burning sensation was the only adverse effect observed.

Although several side effects due to ozone therapy are well known [64], the literature on adverse effects of ozonated oil is very poor. Only a recent study has highlighted two cases of irritant and allergic contact dermatitis that have been caused by topical therapy with pharmaceutical ointments containing ozonated oil [65]. The authors suggested that this could be due to the addition of ozone to olive oil. In particular, in one case, ozonated oil ointment was applied to the lips, which is actually discouraged by the Medication leaflet as the stratum corneum is thin in lip mucosa and the lips are more sensitive to the irritating action of ozone [65].

Topical Oleozon^®^ can be considered an antifungal medication in the treatment of onychomycosis and shows better therapeutic effects than topical ketoconazole. It yields no risk of systemic adverse effects and drug interactions, unlike oral antifungal drugs, and is a low-cost therapy with a mechanism that is probably similar to that observed in bacterial cells [66].

Moreover, several kinds of cosmetic formulations, including cream, shampoo, bath gel, aftershave, body lotion, emulgel, toothpaste, makeup remover, intimate wash, and lip balm are now being marketed, and highlight the presence of an ozonated oil among the ingredients.

Besides being used as cutaneous medication, ozonated vegetable oils are also seeing use for other topical routes of administration, showing the versatility of their applications. The main application fields are dental [67], oral, gynecological, and ophthalmologic.

In the 1930s, E.A. Fisch proposed ozone for use in dentistry to aid in disinfection and wound healing because of its antimicrobial [68], biocompatible and healing properties. In recent years, a number of therapeutic protocols have been developed with ozone to address dental infections associated with periodontal disease and to control infectious oral microorganisms in dental plaque [10]. In fact, ozonated oils, such as sunflower, olive and groundnut, have been employed as intra-canal dressing, reducing the marked anaerobic odor emanating from carious and infected teeth. They have also been used in acute necrotizing ulcerative gingivitis. A new ozonated olive oil, O-zone gel (Alnitec, Cremosano, Cr, Italy), has bactericidal and fungicidal properties. It has been suggested for used in gingivitis and periodontal treatments (for root canal disinfection during endodontic therapies) and for the therapy of early caries.

Moreover, a gel preparation of ozonated olive oil has been added to a standard oral hygiene regimen and was found to reduce enamel demineralization around orthodontic brackets during orthodontic treatment [69]. Another gel preparation, O-zone gel, was compared with two chlorhexidine digluconate (CHX) based agents. It showed a relatively moderate antiseptic effect, which was more evident in the case of Gram-negative bacteria than Gram-positive ones, and inhibited the formation of dental plaque. However, it demonstrated lower antibacterial activity than the CHX-based agents that were tested [70].

In 2011, El Hadary and co-workers proposed the topical application of ozonated plant oils with the short-term subcutaneous co-administration of the immunosuppressive agent cyclosporin A as a strategy to improve osseointegration. In fact, the formation of mature bone was observed to be accelerated around the dental implants [71].

The therapeutic applications of ozonated oils also include the management of oral lesions and conditions. A recent clinical trial has evaluated the efficacy of ozonated olive oil for the treatment of aphthous ulcerations, herpes labialis, oral candidiasis, and angular cheilitis. The study highlighted that the lesions regress in all the patients, although this occurs over variable time periods. In addition, the subjects that suffered from oral lichen planus reported a dramatic reduction in their burning sensations [72].

Another interesting field of application for ozonated oils is gynecology, in particular in the treatment of some pathologies of the vaginal mucosa. In fact, the majority of women contract a number of infections due to sexual behavior and even the physiological hormonal changes that occur during menopause. In particular, vulvovaginal infections caused by *Candida* spp. are the most common infections of female genital systems [73].

Due to the prolonged therapy required for *Candida* spp. and its resistance to conventional treatments, ozonated vegetable oils offer some advantages to fungal infection healing, including low costs and the absence of side effects, consequently, there is a significant benefit for the health of society. Indeed, olive oil itself showed interesting antifungal activity, which is probably due to the inhibition of elastase, an enzyme involved in fungal virulence, by some aliphatic aldehydes components [74].

Rodriguez et al. reported the effectiveness of ozonated oil on *Candida albicans* as early as 1990 [75]. About a decade later, studies comparing ozonated olive and sunflower oils demonstrated that they had similar antimicrobial activity, with low MIC ranging from 0.53 to 0.2 µg/ml, and also detected in vitro activity on filamentous fungi, such as *Aspergillus fumigatus*, and dermatophytes (*Epidermophyton floccosum*, *Microsporum canis*, *Trichophyton rubrum*) [53]. An Iranian research team has recently performed a study comparing the efficacy of ozonated olive oil and clotrimazole cream in the treatment of vulvovaginal candidiasis. The female patients were enrolled and randomly assigned to one of the two groups to receive different treatments. They were evaluated in interviews and a paraclinical examination at baseline and post intervention. The questionnaire used to register changes in the symptoms did not report significant differences between the two groups in their effects on itching and leucorrhea. However, the negative laboratory culture confirmed the activity of both treatments against vaginal candidiasis. Nevertheless, clotrimazole decreased the burning sensation more significantly than the ozone-based treatment [73].

In ocular treatment, ozone can exert anti-inflammatory and bactericidal activity in some pathologies of the eye’s anterior segment and promote regeneration and tissue repair. However, ozonated oil is highly irritating to the corneal tissue. This drawback has been overcome by developing a specific formulation for ophthalmic use, both in animals and humans, that is based on ozonated liposomal sunflower oil plus hypromellose (Ozodrop^®^, FB Vision, Ascoli Piceno, Italy), which is well tolerated and biocompatible with the delicate tissue of the ocular surface. The most common eye disorders encountered in animals [76], such as conjunctivitis, keratitis, keratoconjunctivitis sicca, and corneal ulcers, share some symptoms with human conditions, such as redness, chemosis and exudation. These inflammatory anterior segment diseases necessitate adequate anti-inflammatory therapy. The liposomal formulation is a valid and suitable alternative thanks to its versatility in the management of external ocular disease. In fact, applying Ozodrop^®^ 3–4 times a day controls the pathology until recession. However, the topical administration of a collyrium (1–2 drops per eye every 4 h) needs to be more frequent and repeated to achieve clinical resolution and some patients suffer from a lack of compliance [76,77]. Furthermore, the ozone-based eye drops could be used as preparation for intra- and extra-ocular surgical procedures (i.e. cataract surgery or intravitreal injection) in addition to their antimicrobial properties.

In the veterinary field, ozonated vegetable oils may also be a new means to prevent and/or treat mastitis. They have shown an in vitro effect against the three main bacterial strains that are responsible for mastitis (*S. aureus*, *E. coli*, and *S. uberis*). A solution (post-dipping) enriched with ozonated sunflower oil, and used with a sanitizer to protect the nipples of lactating animals is currently available. The film that is generated completely wraps the nipple without dripping, ensuring practical use. Ozonated sunflower oil helps to keep the tissues intact during milking thanks to its healing properties [44].

Ozonated oil can also inactivate vegetal viruses via the oxidation of specific viral receptors in cellular plants, but a higher dosage is required than for bacteria [42].

It is interesting to underline that many authors correlate the antimicrobial activity of ozonated oils to the peroxide index (PI), believing that activity is greater when PI is higher. However, many investigations have demonstrated that an increase in PI does not affect oil activity [20,78], probably because the antimicrobial mechanism of ozonated vegetable oils is related to oxidizing species, as reported by some authors [19,41].

## 7. Ozonated Oil Delivery Systems: State of the Art and Perspectives

The interesting results obtained by ozonated oils in topical applications has generated, as mentioned above, several commercial formulations. Nevertheless, some aspects can be improved, such as the complete characterization of clear physico-chemical properties (e.g. as described in the previous chapter) to permit fully rational applications.

First of all, research into the antimicrobial activity of these products has been hampered by the lack of standardized and reliable in vitro screening methods. The major difficulties associated with the screening of these products are oil viscosity, which creates difficulties in obtaining stable oil dispersions in aqueous media and the diffusion of lipophilic oil components through agar. Indeed, in antimicrobial tests, dilution in a DMSO solution or the addition of a surfactant (such as Tween 80 (2%) are required to achieve a stable oil emulsion in saline [35,51].

Another issue is the unpleasant smell of ozonated compounds, which is similar to rancid fat, and ozone itself. This opens the way to the search for stable flavoring and inert agents to add to formulations. More importantly, the majority of known ozonated oils are applied to the skin on a regular basis, and their high ozone concentration causes the overstimulation of cells, producing undesired effects such as irritation, rashes and even rapid flaking of the skin [49].

Production and in use tests have reported some issues that are related to the high viscosity of concentrated ozonated oil solutions and stability (only in cold conditions) both for active ingredients and in final cosmetic formulations. Formulations are usually diluted 10 to 20 times during the process.

Delivery systems are engineered technologies that are used to carry active components, improve patient compliance and allow for controlled or targeted delivery [79]. It is known that human skin acts as a barrier against the permeation of exogenous molecules in topical cutaneous use. Delivery systems may enhance the permeation of the active ingredient through the skin layers, controlling its concentration in the formulation and in the skin.

From an industrial point of view, similar formulations can lead to the manufacture of medicinal products, medical devices and cosmetics, which are each controlled by mandatory regulatory constraints. For cosmetic purposes, keeping the active ingredient in the superficial skin layers and avoiding systemic absorption is a major concern. On the other hand, in medicinal products, pharmacological, immunological, and metabolic actions can take place both on the first (skin) layer, in deep layers and include systemic absorption. A medical device does not achieve its principal intended action on the human body by pharmacological, immunological, or metabolic means.

The necessity to guarantee uniform cosmetic products that are sensorially attractive for the consumer and that have long-term stability are essential requirements. The reproducibility and cost-effectiveness of industrial processes are key characteristics for all products types, but are particularly relevant for cosmetics. Quality, safety, and efficacy are always mandatory for a medicinal product. Regarding the safety of a chemical substance, it is relevant to consider that Europe REACH regulation has registered ozonated olive oil with a Regulatory List Number EC No.: 919-979-9 and ozonated sunflower oil (also named Bioperoxoil^®^, Oleozon^®^) with EC No.: 924-751-7. For a classification based on the concentration of ozonated oil contents and their applications, we can report a classification by O’Malley: 1%–2% for lip balm, 2%–3% as moisturizer, 3%–4% for cleanser and anti-aging products, 4%–5% for sport massage oil and, at least 5%–6% for a medical device or pharmaceutical products [80].

The main types of advanced formulations used to increase ozonated oil activity and reduce side effects are reported in the following section. Mainly emulsions (with different droplet sizes) and encapsulation techniques have been proposed so far.

### 7.1. Ozonated Oil-Based Emulsions

Coarse emulsions are colloidal dispersions, characterized by a size range of 1 to 5 μm, that are composed of oil, water and emulsifiers or surfactants. These latter ensure the stability of the oil droplets dispersed in water or vice versa. Pharmaceutical and cosmetic emulsions can be proposed as ointments, creams, gels, and lotions. While ointment formulations are virtually water-free, creams can contain high amounts of water (hydrophilic creams); oil is the dispersed phase and water the continuous (O/W type emulsions). Gels, or better emulsion gels, are spreadable transparent formulations that are obtained via the addition of polymeric gelling agents. Lotions are free flowing creams (mainly of the O/W type) at room temperature. Evaluating the commercial products and literature, gel is the most frequently reported formulation for ozonated oil coarse emulsions.

A dermatological gel containing 30% ozonated sunflower seed oil characterized by 1.60% acidity as oleic acid, 335 meq O_2_/kg peroxide value and 193 mPas viscosity (Oz.Or.Oil 30, CR Cosmetici Srl, Otranto, Italy) fortified with alpha-lipoic acid and Vitamin E (α-tocopherol), has been described by Serio et al. [61]. The same author described preliminary clinical trials in a pilot study on bedsores (at stages I and II) [81]. An efficacy analysis reported that Oz.Or.Oil 30 showed much higher efficacy than the control product (commercial dermatological gel), probably due to the high antibacterial and germicidal power of ozonated oil. Nevertheless, patients indicated the strong smell of Oz.Or.Oil 30, its oily consistency and a slight burn on the skin at the time of application, but also referred that the relief of their symptoms was so rapid that they were pleased with the treatment.

In another invention, an oleogel with high viscosity was directly produced from the ozonization of vegetable oil in the presence of 9% water. This product has been used as a base for pharmaceutical formulations with excellent antimicrobial properties as well as showing skin regeneration and tissue repair [82].

An ozonated oil dual-continuous-phase emulsion has been described by Zhou [83]. Every 100 g of the medicament was prepared from 0.1 to 30.0 g of ozonated oil, 20.0 to 50.0 g of emulsifier, 5.0 to 10.0 g of co-emulsifier, 1.0 to 5.0 g of stabilizer and water. Meanwhile, the invention further includes a preparation method and the application of the ozonated oil dual-continuous-phase emulsion. The ozonated oil emulsion can be diluted with water and oil, can be applied for the prevention and treatment of an animal bedsore disease, showing remarkable effects.

### 7.2. Micro- and Nanoemulsions

There has been increasing interest in using colloidal dispersions to load, protect and deliver active lipophilic components, such as nutraceuticals, drugs, vitamins, antimicrobials, and antioxidants from a range of industrial fields [84]. The terms micro- and nanoemulsions define colloidal oil in water dispersions with size, and thermodynamic and kinetic stability. These are widely used to encapsulate active substances as delivery systems for lipophilic components. Micro- and nanoemulsions are mainly composed of oil, water, surfactants and possibly a co-surfactant. Furthermore, it may be necessary to mechanically agitate or heat the system in order to prepare a micro- or nanoemulsion from the components. Usually, the average droplet size is between 100 and 500 nm. Typically, a greater surfactant-to-oil ratio is required to prepare a microemulsion than a nanoemulsion. The use of more than 20% surfactant will provide a microemulsion, which is more thermodynamically stable, in which the oil molecules may be incorporated between the surfactant tails and/or within the core of the micelle [85]. Regarding the native antimicrobial activity of nanoemulsions, it is worth mentioning that, when the droplets of emulsion can interact and fuse with lipid bilayer of the cell membranes, this fusion is increased by the electrostatic attraction between the anionic charge on the pathogen and the cationic charge of the emulsion. The energy reserved in the oil and detergent emulsion is then released, destabilizing the lipid membrane of the pathogen. This non-specific activity allows for a broad spectrum of activity, and some emulsions are thus suitable candidates for wound healing and surface decontamination [86,87]. Furthermore, increased safety levels and a lack of skin and mucous membrane irritation have been demonstrated.

Micro and nanoemulsions can be also used in biofilm agents for antimicrobial activities [88,89], and the use of nanoemulsions to control the adhesion of biofilms, based on cariogenic bacteria on the tooth surface, is a rational approach to preventing this common oral disease.

An interesting formulation study has recently described nanoemulsion preparation using ozonated olive oil [43]. The oil had an acidity of 36.07% (oleic acid), a peroxide value of 247.01 meq O_2_/kg and iodine value 10.21 (g of I_2_ per 100 g). ln the presence of the surfactant Tween 40, nanoemulsion droplets with a 212.7 nm size were prepared using the emulsion inversion point method, a surfactant-to-oil ratio of 2 and an oil ratio of 2.5%. The preparation demonstrated stability over 30 days of storage at room temperature and the ozonide structure was successfully retained during this time. In vitro results showed that ozonated oils retained their antibacterial activity. This aspect makes it a suitable ozone carrier for various biomedical applications.

Carocci et al. have recently described, in a patent [90], an invention in which ozonated sunflower oil is loaded in a microemulsion where C10-C16-alkyl-trimethyl ammonium chloride and guar hydroxypropyltriammonium chloride are the microemulsion coating and PEG-30 dipolyhydroxystearate is the oily surfactant. No-cytotoxicity was found in cultures of fibroblast L929 for the freshly prepared microemulsion and after one year of storage at room temperature, unlike the same preparation in the form of a gel (5% w/w ozonated oil).

Regarding the development of cosmetics, Antonaccio has described an invention in which a preparation with sunflower oil containing 0.5%–1% of ozonated fraction was emulsified with phasphatidylcholine, carnitine linoleate and. It was claimed that the preparation demonstrated topical active action, such as lipo and cellulitis reduction [91].

### 7.3. Macro-, Micro-, and Nanoencapsulation of Oils

Encapsulation technology may be a feasible option to reduce the undesirable effects of ozonated oils, which include low shelf-stability and sensory properties, and improve the acceptability of oils and products fortified with oil products. Encapsulation is defined as a process in which droplets of the bioactive oil are surrounded by a coating, or embedded in a homogeneous or heterogeneous matrix, to give small capsules with many useful properties [92,93]. In fact, the encapsulation of bioactive oils is a feasible and efficient approach to modulate their release, increase physical stability, protect from oxidation reactions with the environment, decrease volatility and improve patient compliance.

The principal encapsulation methods can be classified as: microparticles, in which oils can be coated by a matrix wall (core-shell structures); nanoparticles, with a size range of 1–800 nm; micelles which possess a hydrophobic central core and a hydrophilic crown; lipid nanoparticles with solid matrix; solid lipid nanoparticles (SLNs) and nanostructured lipid carriers (NLCs), and; liposomes characterized by a lipid bilayer formed with phospholipids of amphiphilic nature. Other encapsulation techniques involve cyclodextrin inclusion complexes and dendrimers, which are polymeric structures with highly branched three-dimensional architectures.

Remarkable uses of nanotechnology can be found in the field of antimicrobial agents for textiles [94]. This is primarily done via the application of inorganic nanoparticles (silver, copper, zinc) to fibers and fabrics. This can produce beneficial innovative active textile products, some of which can be classified as medical devices, such as wound dressings, with the aim of moving research towards the study of novel antimicrobial agents with a wide activity spectrum that avoid resistance.

The micro-encapsulation of ozonated red pepper seed oil has been proposed by Özyildiz et al. [95] with applications in nonwoven fabric to produce a disposable antimicrobial textile material. Ozonated oil was micro-encapsulated via a complex coacervation method (that can be induced in systems containing cationic and anionic hydrophilic colloids) using gelatin and arabic gum as well as materials in the presence of a surfactant agent.

The ozonation reaction in red pepper seed oil, which has a high degree of unsaturation, occurs mainly through linoleic acid and oleic acid. Batches of particles with a range 19–37 μm and with oil loading of 47%–56% were obtained (no data on acidity, peroxide value and iodine index were reported). Antimicrobial activities against *E. coli*, *P. aeruginosa*, MRSA, *C. albicans*, and vancomycin-resistant *E*. *faecium* have shown that the encapsulated ozonated oil retained its efficacy in the microcapsule form. The antimicrobial effect can be explained by the presence of anti-microbial compounds, such as aldehydes and carboxylic acids (e.g. azelaic and pelargonic acids), formed by the ozonation process. Fabrics impregnated with 20% to 30% active microcapsules were also found to be very active against antibiotic-resistant test microorganisms. Furthermore, the observed prolonged activity was found to be important for the production of functional textiles.

Medical textiles with antimicrobial and wound healing properties have also been studied by Besen et al. [96]. For this purpose, St. John’s Wort oil and flax seed oil were ozonated (final acidity 21.02% as oleic acid), and the oils were encapsulated with arabic gum via the simple coacervation method (arabic gum is the wall-forming material and protein precipitation was induced by a pH change in the reaction medium). Oil loading was 53% with a particle size of 19 μm. The antibacterial activity of the raw oils was assayed against *E*. *coli* and *S*. *aureus*. While the raw oil had low antibacterial activity, the ozonated oils showed a high inhibition zone (high activity). This activity can be explained by the contents of the ozonide structures.

Using the in vivo circular excision wound rat model, it became clear that the raw oils had wound healing properties and that their wound healing activity increased after the ozonation process. Fabrics that include microcapsules of ozonated oils have been found to display higher % reductions in wound area values than negative controls, raw fabric and microcapsules containing raw oils after 10 days of use. While skin irritation and sensitization were absent, mild to moderate cytotoxicity was observed.

The microencapsulation of ozonated oils has also been reported in a patent that was filed by O’Malley for use in oral care products. Micro-encapsulated ozonated oils were proposed for incorporation into dental floss, tooth brush bristles, chewing sticks, comforters, denture bases, plastic retainers, and orthodontic devices [80].

The co-administration of multiple nanoparticles and ozonated oil has been reported by Elshinawy et al. [97]. Synergic activity between ozonated olive oil, in the presence of chitosan, and silver nanoparticles was used to prevent biofilm formation and eradicate resistant endodontic pathogens from root canals. Bacterial biofilms generated by *S. mutans*, *E. faecalis*, and *C. albicans* were used in ex vivo tests. Chitosan nanoparticles reduced single and mixed species biofilms by 97% and 94%, respectively. Furthermore, ozonated oil exerted an 86% and 79% reduction in biofilms, respectively. The combination of ozonated oil/chitosan nanoparticles worked the best and exerted a significant killing effect in the form of a 6-log reduction in viable cells. Nevertheless, ozonated oil demonstrated its safety by showing the least cytotoxicity in human fibroblasts. In addition to the antimicrobial activity of the oil, caused by the ozone-derived compounds, chitosan antibiofilm activity can be attributed to its ability to penetrate and damage biofilms thanks to its cationic properties.

Liposomes are among the most intensely studied particulate colloidal systems. Their attraction lies in their composition, which makes them biocompatible and biodegradable. They consist of an aqueous core that is entrapped by one or more bilayers, which are composed of either natural or synthetic lipids. Liposomes composed of natural phospholipids are biologically inert and weakly immunogenic, and possess low intrinsic toxicity. Liposomes have been predominantly used for parenteral administration and many formulations have been commercialized, including Ambisome^®^ (amphotericin B) or Doxil^®^ (doxorubicin). Liposomes are able to reduce the side effects, improving activity, of the systemic delivery of potent anticancer drugs [98,99].

Vegetable essential oils with antimicrobial activity that are loaded into liposomes have been widely reported, while liposomes containing ozonated oils have only recently been described by Spadea et al. [76]. A liposomal sunflower ozonated oil plus hydroxypropylmethyl cellulose (Ozodrop^®^, FB Vision, Ascoli Piceno, Italy) was formulated as collyrium (details and characterization not available). The preliminary results demonstrated that ozone-based eye drops have anti-inflammatory and bactericidal activity, in addition to promoting tissue repair, and can be useful for the management of external ocular pathologies in both animals and humans.

More recently, the cytotoxicity of liposome-vehiculated ozonated oil against *S. aureus* and *P. aeruginosa*, which are among the most frequent eye pathogens, has been analyzed [100]. The microbiological results clearly show antimicrobial efficacy and there was no evidence of any toxicity against keratinocytes.

Cyclodextrins (CD) are glucose-based oligosaccharides that are capable of interacting with small and hydrophobic molecules, forming inclusion complexes. These complexes provide the molecules with enhanced solubility and physicochemical stability. A reference paper evaluating the interaction between CD and lipids, the inclusion mechanism and their uses in emulsion preparations has been written by Duchêne et al. [101]. The formation of inclusion compounds with fatty acids occurs through the creation of hydrogen bonds between the carboxyl of the fatty acid chain and the hydroxyls at position 6 of the cyclodextrin and the creation of hydrophobic interactions between the fatty acid hydrocarbon chain and the cyclodextrin cavity. C12 fatty acid chains are the minimal length needed to obtain the surface-active role of cyclodextrins, and the stoichiometry of inclusion compounds between alpha or beta cyclodextrins and C_12_–C_18_ fatty acids ranged between 1.7 to 3.1. Fatty acids and monoglycerides lead to water-soluble inclusion complexes, while complexes with triglycerides are water insoluble. Alpha- and beta-cyclodextrins allow optimum molecule organization and interactions between the soybean triglycerides and the cyclodextrin cavities to occur. The use of cyclodextrin in emulsion formulations is interesting because it allows the high amount of classical surfactant necessary to stabilize the simple internal emulsion to be decreased.

The inclusion of ozonated oils in cyclodextrin has recently been reported by Beşen et al. [102]. The aim of the study was to apply antibacterial finishing to cotton fabrics using a β-CD inclusion complex with ozonated olive oil to produce a disposable surface without the characteristic ozone odor. The ratio of ozonated oil to β-CD was kept at 1:6 (v/v) for inclusion complex preparation. Solutions of olive oil (10%) in ethanol and β-CD (10%) in ethanol:water (1:2) were prepared separately and stirred using a magnetic stirrer. The oil solution was added dropwise into the β-CD solution while stirring, and the solution then was kept in the refrigerator for 20 h, and the inclusion complex precipitate was then filtered and dried. However, a precise determination of the amount and the preferential inclusion of some of the molecules produced in the ozonization process were not described. The authors observed, in the TGA evaluation results, that thermal stability increased when the ozonated oil was sequestered in the inclusion complex. The malodor of ozonated oil was completely removed.

## 8. Future Perspectives and Conclusions

As described above, several strategies have been proposed to increase the efficacy, and expand the physico-chemical and sensorial characteristics, of products containing ozonated oils. Nevertheless, to further improve penetration into affected topical areas, and thus reduce the dose required and side effects, one can exploit the great work done and lessons learned in previous research, in particular in nanotechnological approaches, on oil or highly lipophilic agents for topical delivery. When evaluating other possible formulations for ozonated oil, it is necessary to bear in mind the scalability, manufacturing cost and the safety of other added components (the best raw materials belong to the Generally Recognized As Safe (GRAS) category as they have biocompatibility or toxicity-associated problems) and, of course, no reactivity against trioxolane and oxidized derivatives of fatty acids should be observed. The research into lipid-based delivery systems indicates that emulsions (microemulsions in particular) must surely be profitable. In particular an interesting exploration field may be represented by multiple emulsions formulation, because this technique permits different substances, both hydrophilic and lipophilic, to be delivered and protected.

Multiple emulsions, also described as “emulsions of emulsions”, are complex poly-dispersed systems, in which the dispersed phase is itself an emulsion present as fine droplets. Two types of multiple emulsions are: Water-in-oil-in-water (W/O/W) and oil-in-water-in-oil (O/W/O). However, very interesting colloidal systems may only be suitable for use when instability-related problems are solved. In the case of ozonated oils, the increased viscosity of oil can improve microemulsion stability. To further increase this characteristic, a strategy to cover emulsion droplets with viscous lamellar liquid crystal, which extends throughout the continuous phase, can be proposed. The nature of the oily phase and the surfactants used to ensure the formation of these liquid crystal structures is of central importance. Several kinds of emulsifiers can be employed in combination, while the preparation method sometimes requires heating and high shear mixing [103]. Liquid crystalline systems formed with olive oil have recently been described. The surfactant sorbitan monooleate ethoxylated has been combined with sorbitan monooleate 20 EO in a 1:3 ratio and was used at a concentration of 70% to 80% of total mass [104]. Furthermore, lyotropic liquid crystals can also be usefully employed as mucoadhesive delivery systems, providing longer retention times at the application site. An example of the topical administration of liquid crystalline systems is found in the work of de Araújo et al. on the vaginal administration of drugs [105]. This method may be also suitable to delivery oils and drugs at the same time.

Continuing the evaluation of lipid-based carrier systems, it is interesting to cite the evolution of liposomal technology, which is represented by nanostructured cochleate. Cochleates are spiral structures that can bear a drug incorporated into the lipid bilayer and that have positive charges on the outer surface. The presence of anionic lipids, such as phosphatidylcholine, in liposome preparation is followed by nucleation and aggregation processes that are induced by the addition of divalent cations, such as Ca^2+^, Mg^2+^, and Zn^2+^, in the presence of a mixture of water and ethanol. The topical administration of an antifungal agent with improved activity has been described [106]. Although showing advantages, such as lower susceptibility to oxidation, the use of cochleate is restricted due to disadvantages such as some instability and aggregation during storage conditions and higher manufacturing cost [107].

The use of lipid nanoparticles, such as SLN and NLC, is another area that has not yet been explored for ozonated oils. The topical application of these lipid nanoparticles on the skin creates a stable monolayer because of their adhesiveness properties. The hydrophobic monolayer has an occlusive effect that prevents water evaporation, allowing a potentiation of local antibacterial activity while supporting the skin’s moisture level. Both SLN and NLC also display good biocompatibility and tolerability and are currently widely used in skincare products [108,109,110].

Over the last decade, microsponge technology has emerged as attractive option for topical dermatological treatment [111]. This colloidal delivery system has a typical size range of 5–300 μm meaning that its passage through the stratum corneum is hindered. The sponge-like carriers are composed of polymers that are dispersed in an aqueous system. Several polymers have been proposed for the formation of microsponge ‘cages’, including Eudragit (polymethacrylates), polylactide-co-glycolic acid, polydivinyl benzene, ethyl cellulose. Formulations as gel, emulgel, ointment, and cream incorporating microsponges have been widely described. [112,113] Their features have been found to enhance the loading ability and stability of active components, and reduce local irritation, meaning that this technique is used in cosmetic and dermatological products. Essential oils with antibacterial activity [114,115] and drugs [116,117,118] have shown interesting results in topical delivery, when loaded into microsponges, over different wound infected models. Other potential topical applications are ocular use [119], and antifungal vaginal formulations [120].

Intensive research into polymer applications in cosmetics has suggested other sophisticated encapsulation systems [121]. Furthermore, the release of oil can be also obtained in silica-based systems, such as hollow mesoporous silica spheres [122].

In conclusion, ozone has provided a great deal compelling evidence for its use in topical administration and has an appropriate safety profile, especially when administered as ozonated oil. Ozonated oils have demonstrated activity and can be proposed for use in the prevention and treatment of chronic local infections, in appropriate formulations and in controlled cases, as an alternative to topical antimicrobial agents. This can be useful as an alternative to contrast the widespread and indiscriminate use of topical agents, particularly mupirocin and fusidic acid, which has led to the emergence of bacterial resistance, predominantly in staphylococci [123]. Nevertheless, as suggested by the Cochrane report on the meta-analysis evaluation of treatment foot ulcers in patients with diabetes: “High quality randomized controlled trials with longer duration of follow-up are needed to determine the effectiveness and safety of ozone therapy” [124]. Nevertheless, work in this field should include trials that perform head-to-head comparisons of topical antimicrobials and ozonated oils and consider clinical outcome and bacterial resistance. At the same time, intense chemical and technological characterization is needed to allow comparisons of the proposed formulations. In particular, the structural and isomeric composition of secondary ozonides, which are generated during ozonation processes, makes investigations into the role of stereochemistry highly significant when the possible therapeutic effects of ozonated oils are evaluated. Furthermore, the use of protic solvents during ozonation should be carefully considered in order to assess the possible presence of hydroperoxide derivatives. In the future, it would be desirable to advance formulations that are characterized by optimized skin permeability, safety in open wounds and increased binding to microorganism membranes in order to foster disinfecting activity and reduce exposure time.

## Figures and Tables

**Table 1:** Chemical and physical parameters of ozonated oils.

Vegetal Oil	Analyzed Physico-Chemical Parameters	Reference
Olive oil	Peroxide value Acidity value Iodine value p-anisidine value Viscosity pH	[42]
Olive oil Sunflower oil	Peroxide value Acidity value Iodine value	[34]
Flaxseed oil Sunflower oil Baru oil Oleogel (from 24 h of ozonolysis in the presence of water)	Peroxide value Acidity value Iodine value	[41]
Sunflower oil ozonated using medical oxygen Sunflower oil ozonated using air	Peroxide value Acidity value Iodine value Moles of double bonds Viscosity Density	[19]
